# Heterologous DNA prime-protein boost immunization with RecA and FliD offers cross-clade protection against leptospiral infection

**DOI:** 10.1038/s41598-018-24674-8

**Published:** 2018-04-24

**Authors:** Veerapandian Raja, Sankaran Sobana, Charles Solomon Akino Mercy, Bianca Cotto, Durlav Prasad Bora, Kalimuthusamy Natarajaseenivasan

**Affiliations:** 1 Medical Microbiology Laboratory, Department of Microbiology, Centre of Excellence in Life Sciences, Bharathidasan University, Tiruchirappalli, 620 024 Tamilnadu India; 2 Lewis Katz School of Medicine, Temple University, Philadelphia, PA 19140 USA; 3 Department of Microbiology, College of Veterinary Science, Assam Agricultural University, Guwahati, 781022 Assam India

## Abstract

The emergence of >300 serovars of *Leptospira* confounded the use of generalized bacterin, the whole cell lysate, as vaccines to control leptospirosis. Because of substantial genetic and geographic heterogeneity among circulating serovars, one vaccine strain per serovar cannot be efficacious against all the serovars. We have performed heterologous DNA prime-protein boost vaccination challenge studies in hamsters using *in vivo* expressed, leptospiral recombinase A (RecA) and flagellar hook associated protein (FliD). We prepared the monovalent recombinant protein, plasmid DNA, and DNA prime protein boost adjuvant vaccines. The whole cell bacterin served as a control. Our data show that (i) RecA and FliD have multiple immunogenic B and T-cell epitopes with highly conserved domains among most prevalent pathogenic *Leptospira* spp., (ii) humoral and cell mediated immune responses were induced remarkably, (iii) provides significant protection against homologous (Autumnalis strain N2) and cross-clade heterologous (Canicola strain PAI-1) challenge infection for the heterologous prime-protein boost (∼91–100%) and, the DNA vaccine (∼75–83%). Recombinant protein vaccine shows only partial protection (∼58–66%), (iv) RecA prime-protein boost vaccine shows sterilizing immunity, with heterologous protection. This RecA/FliD prime-protein boost strategy holds potential for vaccination against animal leptospirosis and for a better control of zoonotic transmission.

## Introduction

Leptospirosis remains a major cause of mortality and morbidity worldwide, with almost 1.03 million cases and 58,900 deaths annually^1^. It is also reported as an important public health issue in Southeast Asia, Oceania, Caribbean, Andean, Central and Tropical Latin America, and East Sub-Saharan Africa^1–3^. The symptoms of leptospirosis vary from mild flu-like illness to multi organ failure and in advanced cases leads to death of the infected host^4^ emphasizing the importance and impact of the disease burden worldwide.

Whole-cell leptospiral vaccines are available and used to protect against leptospiral infection^5^. But, the lack of cross-serovar protection preceded the development of subunit vaccines, including recombinant protein and DNA vaccines. Several leptospiral surface antigens were proposed as efficient vaccine candidates in experimental animal models^6^. Nonetheless, they still have limitations such as the inability to induce cross protection among pathogenic serovars and generation of short-term immunity^7,8^. These shortcomings prevent them from being effective and reliable vaccines for different geographic areas that have variations in serovar distribution^9^.

The genetic immunization (by DNA vaccines) could induce both humoral and cellular immunity, persistent expression of heterologous antigen, and a memory response against the infectious disease. Despite these advantages, the major limitation of DNA immunization is its poor immunogenicity^10^. To overcome the limitations, over the past decade, studies have shown that prime-boost immunizations can be given with unmatched vaccine delivery methods while using the same antigen, in a “heterologous” prime-boost format^11^. Such heterologous prime-boost can be more immunogenic representing a novel way of immunization. With this view point we believe that the DNA prime-protein boost strategy, in which the immune response is primed with a DNA vaccine and subsequently boosted with a protein will constitute a promising approach to improve the efficiency of DNA immunization^11–13^. Additionally, the leptospiral immunoglobulin-like proteins (Lig) evidenced a promising vaccine candidate because of its *in vivo* expressing potential^14^. Apart from Lig proteins, none of the other leptospiral candidates proved its efficacy. Recently, we discovered proteins that are expressed during leptospiral infection (*in vivo* expressed proteins) in the host and these proteins were shown to elicit strong IgM responses for conclusive diagnosis of leptospirosis^15^. Considering the immune response during natural infection among the human cases, evidencing their durable immunogenicity we developed an efficient DNA prime-protein boost strategy with our newly identified proteins RecA and FliD. Our data suggest, the use of RecA/FliD DNA prime boost vaccine broadens protective immune response in hamster against heterologous challenge.

## Materials and Methods

### Bacterial strains and culture conditions

The leptospiral strains used in the present study were cultured in Ellinghausen-McCullough-Johnson-Harris (EMJH) basal medium supplemented with *Leptospira* EMJH enrichment (Difco, BD, Franklin Lakes, NJ, USA) and maintained at 30 °C. *Leptospira interrogans* serovar Autumnalis strain N2 and *Leptospira interrogans* serovar Canicola strain PAI-1, isolated from human urine samples of ailing patients were used^16^ for challenge experiments as per^17^ Forster *et al*.^17^. All procedures with *L*. *interrogans* were conducted using leptospiral MACS (Mouse Adapted Challenge Strain) as per our earlier report^15^. *Escherichia coli* strains like DH5α, BL21 (DE3), and BL21 (DE3) pLysS (Novagen, Madison, Wis.) were routinely grown in Luria-Bertani (LB) medium (Sigma Aldrich, St Louis, MO).

### Conservation and prediction of immunogenic domains

The presence of *recA* and *fliD* among *Leptospira* spp. was confirmed by PCR amplification using genomic DNA from 14 serovars belonging to six species; *L*. *interrogans*, *L*. *borgpetersenii L*. *kirschneri*, *L*. *alexanderi*, *L*. *weilli* and *L*. *biflexa* as described earlier^15^. The coding sequences were aligned using CLC Main workbench (version 7.9.1), based on the ClustalW algorithm. The protein sequence of RecA (AGW25358) and FliD (WP_001110738) were retrieved from GenBank and subjected for B-cell and T-cell epitope prediction using BCPred^18^, NetMHCcons^19^ and NetMHCII^20^. For peptide - MHC binding analysis, a panel of alleles were selected, which account for 97% of HLA allelic variants in most ethnicities. NetMHCcons and NetMHCII were used to predict T- cell specific epitopes. Predictions were made for 8–14 mer peptides of MHC I and 15 mer peptides of MHC II for analysis^8,21,22^. The conservancy of epitopes and antigenicity were determined by Immune Epitope Database Analysis Resource (IEDB)^23^ and VaxiJen score^24^.

### Leptospiral bacterins and recombinant proteins

Leptospiral bacterins, whole cell lysate (WCL) were prepared from *Leptospira interrogans* serovar Autumnalis strain N2 as previously described^25^. RecA or FliD recombinant proteins were overexpressed and purified using *E*. *coli* expression system as described elsewhere^15^. The purified recombinant proteins were dialyzed and the protein concentration determined using a bicinchoninic acid assay (BCA) kit (Sigma, St.Louis, MO). The vaccine formulation was prepared as a proportion of 100 μg of protein in Alhydrogel (Sigma, St. Louis, MO) for first booster on day 0. On day 21, a second booster of 100 μg protein was administered by subcutaneous injections.

### Construction of recA and fliD DNA vaccine

For DNA immunization, genomic DNA was extracted from *L*. *interrogans* serovar Autumnalis strain N2 using the QIAamp DNA extraction kit (Qiagen, Valencia, USA). The primers were designed for full-length leptospiral *recA* and *fliD* according to *L*. *interrogans* serovar Autumnalis strain N2 gene sequence (GenBank accession no. KF240786) using Primer 2 (Scientific & Education Software, 1991). The primer used for *recA* and *fliD* are specified in Table S1. The PCR amplification was performed in 50 μL containing 10X Taq buffer, 1.5 mM MgCl_2_, 200 μM dNTPs, 1.25 U of Dream *Taq* DNA polymerase (Thermo Scientific, Rockford, IL) and pair of primers (each 10 ρM) and approximately 50 ηg of template DNA. The temperature profile for amplification was initial denaturation at 95 °C for 5 min, denaturation at 95 °C for 1 min, annealing at 60 °C for 30 sec and extension at 72 °C for 1 min, for 30 cycles, followed by a final extension at 72 °C for 5 mins. Amplified *recA* and *fliD* were cloned into multiple cloning site of mammalian expression vector pEGFPN3 (Addgene, Cambridge, MA, USA). The identity and orientation of the cloned inserts were confirmed by DNA sequencing (Macrogen, South Korea). *E*. *coli* DH5α competent cells were transformed with the *rec*A-pEGFPN3- or *fliD*-pEGFPN3- construct, and confirmed using REA analysis and DNA sequencing. The constructed plasmids (recA-pEGFPN3 or fliD-pEGFPN3) were purified in large quantity by EndoFree Plasmid Maxi Kit (Qiagen, Valencia, USA). The quality and quantity of the purified plasmids were checked by agarose gel electrophoresis and plasmid DNA quantification using a Biophotometer (Eppendorf, Hamburg, Germany).

### Immunization and challenge experiments

The vaccine preparations used in this study were phosphate buffered saline (PBS), pEGFPN3, bacterin whole cell lysate (Heat killed whole leptospires prepared from 10^9^ bacterial cells), purified rRecA/rFliD, *recA*-pEGFPN3, *fliD*-pEGFPN3, *recA*-pEGFPN3 + rRecA, *fliD*-pEGFPN3 + rFliD. Alhydrogel (Sigma, St. Louis, MO) was used as an adjuvant in all vaccine preparations. 6 or 8 female hamsters aged 4 to 6 weeks were used for immunization and protection experiments.

Hamsters were vaccinated with DNA in two doses (100 μg/dose) by intramuscular (IM) injection. For prime boost vaccination strategy, first dose of 100 µg DNA, followed by the second protein booster of 100 µg was administered. Forty-two days after the first immunization, hamsters were challenged intraperitoneally with 5x median lethal dose (LD50) of *L*. *interrogans* serovar Autumnalis strain N2 (1.37 × 10^7^ leptospires) and *L*. *interrogans* serovar Canicola strain PAI-1 (1.6 × 10^3^ leptospires) as homologous and heterologous infection as described in our earlier studies^26^. Two independent animal cohort experiments were conducted for the evaluation of the vaccination strategy.

Animals were monitored daily and those which survived were euthanized on day 70 after challenge. Blood samples were collected from the retro orbital venous plexus on 14^th^, 21^st^ (before booster) and 42^nd^ days (before challenge), and the sera were stored at −80 °C. The *in vivo* expression of DNA vaccine constructs of *recA* and *fliD* were performed by Western blot analysis^27^ with specific rabbit hyper immune sera^15^. Animal experiments described in this study were carried out in strict accordance with the recommendations approved by the Committee for the Purpose of Control and Supervision on Experiments on Animals (CPCSEA), Bharathidasan University Ethics Committee in Animal Experimentation (BDU/IAEC/2011/29) and Bharathidasan University Institutional Biosafety Committee (BT/BS/17/29/2000 PID).

### Humoral immune response

ELISA was performed to monitor the humoral immune response. Briefly, polystyrene 96 well microtitre plates (Nunc; Thermo Scientific, USA) were coated overnight at 4 °C with 0.2 µg/well of rRecA/rFliD as antigen using carbonate coating buffer (pH 9.6). The plates were washed three times with PBS containing 0.05% [v/v] Tween 20 (PBST) and blocked with 4% non-fat milk in PBST. Hamster sera were added at a dilution of 1:200 for 1 h at 37 °C, followed by three washes with PBST. Goat anti-hamster IgG peroxidase conjugate (Sigma Aldrich, St. Louis, MO) in a dilution of 1:4000 was added and incubated at 37 °C for 1 h and washed five times with PBST. The reaction was visualized with *o*-phenylenediaminedihydrochloride (Sigma-Aldrich, St. Louis, MO). The reaction was stopped by the addition of 0.1 M sulphuric acid (Merk, Germany) and optical density (O.D) was measured at 490 nm (Bio-Rad, Hercules, CA, USA). Mean values were calculated from sera samples assayed in triplicate.

### RNA isolation, cDNA synthesis, and qRT-PCR

Total RNA was isolated from the spleen cells using the TRIzol reagent (Invitrogen, Carlsbad, CA) and cDNA was synthesized using the iScript cDNA synthesis kit (Bio-Rad, Hercules, CA, USA). Quantitative reverse transcriptase PCR (qRT-PCR) was performed using a CFX96 Touch^TM^ Real-Time PCR detection system (Bio-Rad, Hercules, CA, USA). The qRT-PCR using SYBR Green PCR Master Mix (Bio-Rad, Hercules, CA, USA) and primers^28,29^ was carried out in a 25 μL reaction volume (50 ng cDNA, 12.5 μL Master Mix, 0.5 μM of each primer). Primer details are given in Table S2. The cycling conditions consisted of 95 °C for 10 min (denaturation), followed by target DNA amplification for 45 cycles (95 °C for 5 s, 60 °C or 61 °C for 30 s, and a variable extension time at 72 °C). The melting curves were analyzed immediately after amplification at a linear temperature transition rate of 0.1 °C/s from 55 to 95 °C, with continuous fluorescence acquisition. The relative CT (ΔΔCT) method was used to quantify cytokine gene expression^28^. Briefly, the fold change of each target mRNA was normalized to β- actin housekeeping gene CT (ΔCT), and compared to a calibrator sample, the same normalized gene in the pre-immune sera sample (ΔΔCT). The final value represents the mean of triplicate relative fold between immunized and non-immunized hamster.

### Sterilizing immunity by Culture and Real-Time PCR

The animals that survived up to 28 days post challenge were euthanized. Sterilizing immunity was determined by culture isolation of leptospires from kidney tissues as described previously^30^. For quantification of leptospiral DNA, DNA was extracted from freshly isolated kidney tissues from immunized hamsters using DNeasy blood and tissue kit (Qiagen, Valencia, CA). 16 S rRNA gene based real-time PCR was performed on a CFX96 Touch^TM^ Real-Time PCR system (Bio-Rad, Hercules, CA, USA)^29^.

### Statistical analysis

The Fisher exact test and log-rank test were used to determine significant differences in mortality and survival rates, respectively, among the experimental groups. Antibody levels were analyzed with two-way ANOVA to compare differences between the groups. The Student’s t-test was used to determine significant differences among the mRNA relative expression analyses. A *P* ≤ 0.05 was considered significant in all analyses. The data were computed with either GraphPad Prism version 7.0 or SigmaPlot version 11.0. High degree identify templates were used to generate the initial 3D structure of RecA and FliD protein using Modeller 9.19.

## Results

The amplification of *recA and fliD* gene showed the existence of both the genes in all pathogenic leptospiral serovars belonging to five species including *L*. *interrogans* (Autumnalis, Australis, Bataviae, Canicola, Pomona, Icterohaemorrhagiae), *L*. *kirschneri* (Grippotyphosa, Cynopteri), *L*. *borgpetersenii* (Ballum, Javanica), *L*. *alexanderi* (Manhao), *L*. *weilii* (Celledoni). Interestingly, the genes were absent in non-pathogenic *L*. *biflexa* (Andamana, Semaranga) serovars **(**Fig. S1**)**. Additionally, protein blast (BLASTp) analysis showed RecA and FliD proteins to be conserved among all pathogenic leptospiral species **(**Figs S2 and S3**)**.

**Figure 1:**
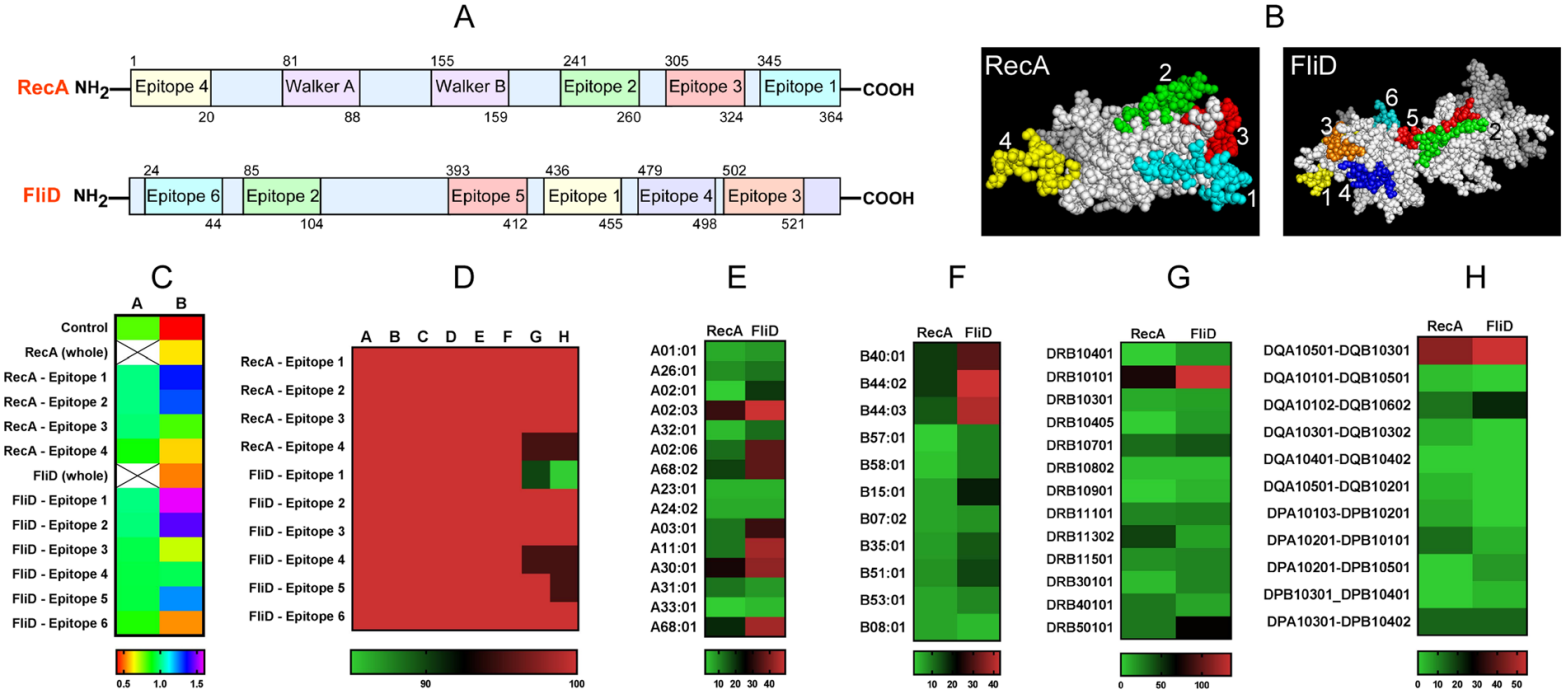
Conservation of the predicted epitopes in RecA and FliD. (**A-B**) The predicted B-cell epitopes were highlighted in sequence alignment as well as in 3D structure of RecA and FliD. RecA contains highly conserved Walker A motif and Walker B motif and FliD contains N terminus region, middle region and (**C**) terminus region. (**C**) Heat map showing predicted B-cell epitopes based on BCPred, conservancy and VaxiJen scores in RecA and FliD. A–BCPred score (Control score -0.8), B–VaxiJEN score (Control score -0.4). Blank or excluded were shown as X through the cells. (**D**) Epitope conservancy: A–*L*. *interrogans* serovar Autumnalis (AGW25358, EMN53408), B–*L*. *interrogans* serovar Australis (EMY22543, OOB99359), C–*L*. *interrogans* serovar Canicola (EKO69724, EKO71148), D–*L*. *interrogans* serovar Copenhageni (AAS70334, AAS69344), E–*L*. *interrogans* serovar Icterohaemorrhagiae (EKP23302, EMO07105), F–*L*. *interrogans* serovar Pomona (EMF34262, EMJ59305), G–*L*. *kirschneri* serovar Grippotyphosa (EJO70816, EJO70003), H–*L*. *noguchii* (WP_004424072, WP_004450466). (**E**–**H**) Heat map showing number of predicted T- cell strong binder major histocompatibility complex (MHC-I and II) epitopes in RecA and FliD. (**E**) List of predicted immunogenic T-cell epitopes for HLA-A allelic variants. (**F**) List of predicted immunogenic T-cell epitopes for HLA-B allelic variants. (**G**) List of predicted immunogenic T-cell epitopes for DQA1/DQB1 and DPA1/DPB1 locus. (**H**) List of predicted immunogenic T-cell epitopes for DRB1/3/4/5 locus.

Because, we saw the proteins to be conserved among all pathogenic leptospires we then asked whether these proteins have immunogenic potential to induce strong B and T cell response. We performed mapping of B and T-cell epitopes of RecA/FliD by BcPred and NetMHCcons and NetMHCII analysis. BcPred analysis showed 4 and 6 immunogenic B-cell specific epitopes for RecA and FliD respectively **(**Fig. 1A and B**)** with VaxiJen score ranging between 0.85 to 0.99 **(**Table S3**)**. The predicted epitopes were conserved among the prevalent pathogenic leptospiral species as seen from the heat map analysis **(**Fig. 1C and D**)**. The alleles selected for binding affinity belong to both MHC I and MHC II alleles. The lists of alleles with strong binder epitopes (IC50 < 50 nM) were considered as potential binders **(**Table S4**)**. The total no of strong binders for RecA and FliD was found to be high among the MHC alleles used in the present study, which account for 97% of HLA allelic variants in most ethnicities.

Among the different alleles A0203, A0206, A1101, DRB0101, DRB0701, IAd, IAs, TAP had the least IC50 value (IC50 value < 50) and considered to be potential binders **(**Fig. 1E–H**)**. Because *in silico* analysis evidenced the immunogenic potential of RecA and FliD proteins, we then asked whether genetic vaccines preparation from these proteins will offer protective immunization against leptospiral infection. We next prepared DNA vaccines by cloning the CDs of RecA and FliD in to pEGFPN3 vector. The cloned *recA*-pEGFPN3 *and fliD*-pEGFPN3 vaccine constructs were confirmed by REA analysis **(**Fig. S4**)** followed by sequencing. For subunit vaccine, the rRecA and rFliD proteins were over expressed in *E*. *coli* as described previously^15^. Bacterin vaccine (WCL) was prepared from *L*. *interrogans* serovar Autumnalis strain N2 and the sterility check showed absence of leptospiral growth in bacterin inoculated EMJH media. All vaccine candidates were given intraperitoneally and the *in vivo* expression of cloned *recA*-pEGFPN3 *and fliD*-pEGFPN3 were monitored by western blot analysis of hamster hind leg tissue lysates probed with the corresponding hyper immune sera. Western blot analysis showed expression of RecA/FliD in hamsters immunized with the corresponding DNA vaccines **(**Fig. S5**)**.

### Humoral immune response

The specific humoral immune response in immunized hamsters was evaluated using an IgG-ELISA with serum samples collected on days 0, 14, 21, and 42 postimmunization (p.i.). To ensure the specific reactivity of the serum samples against recombinant RecA/FliD, we performed an IgG ELISA using unrelated leptospiral proteins rLigA and rLipL32 with N-terminal 6x-His-tag. The sera was highly specific for the recombinant RecA/FliD ruling out the possibility of immunization not against the potentially immunogenic 6x His-tag **(**Fig. S6**)**.

**Figure 2:**
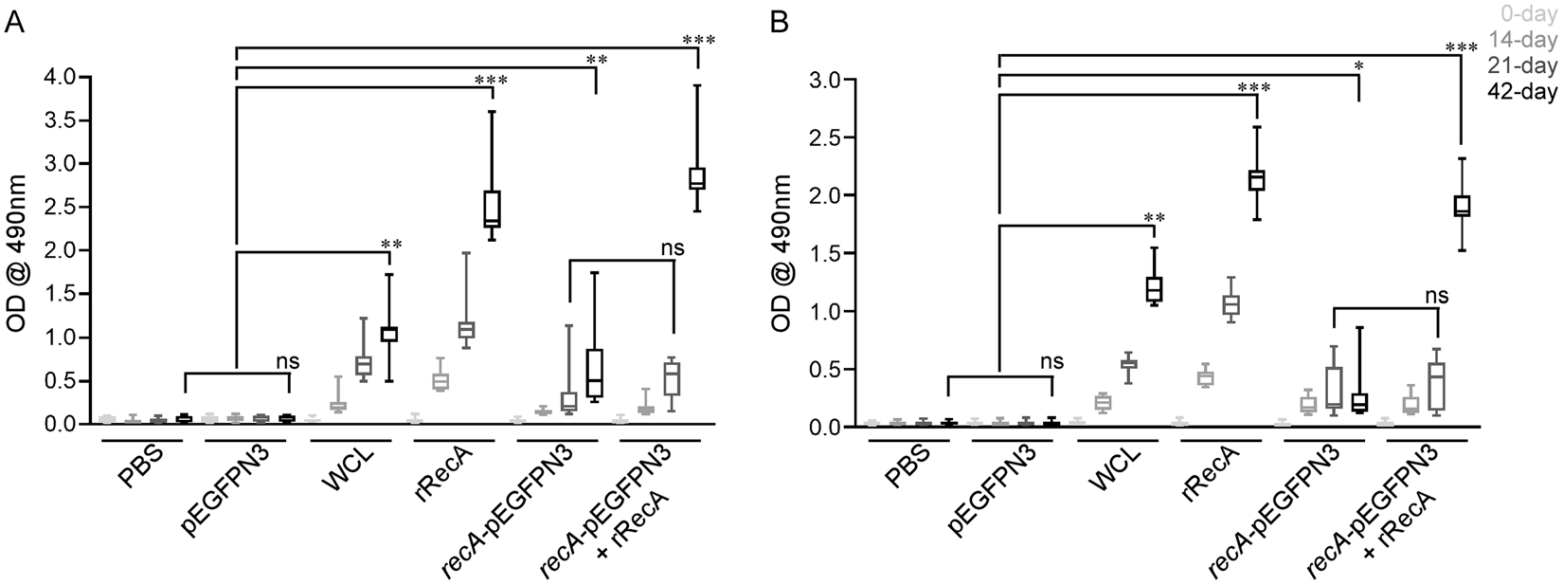
Evaluation of humoral immune response in control and immunized hamster groups by IgG ELISA. (**A**) rRecA antigen used, (**B**) rFliD antigen used. Graphs represent the Mean ± SD of the optical density of the sera obtained on day 0 (pre-vaccination), on 14^th^, 21^st^ (before booster) and 42^nd^ days (before challenge). ns-no significance, *P < 0.05, **P < 0.01, ***P < 0.001. *P* values were obtained through comparison with the negative control (PBS) using a Tukey’s multiple comparisons test by 2way ANOVA.

Significant levels of circulating anti-RecA/FliD antibodies were detected (*P* < 0.001) **(**Fig. 2**)**. At 21 and 42 days p.i., there was a significant induction of humoral immune response in hamsters immunized with the RecA/FliD subunit preparation compared to that in the negative control group (PBS-Alhydrogel/pEGFPN3-Alhydrogel) (*P* < 0.001). The IgG response in hamsters immunized using the prime-boost strategy differed significantly from that in the negative-control group on day 42 p.i. (*P* < 0.001). In the group immunized with the DNA vaccine, there were detectable levels of IgG, but this was found to lower even after 42 days p.i (*P* < 0.05) **(**Fig. 2**)**.

### Cytokine expression profile

**Figure 3:**
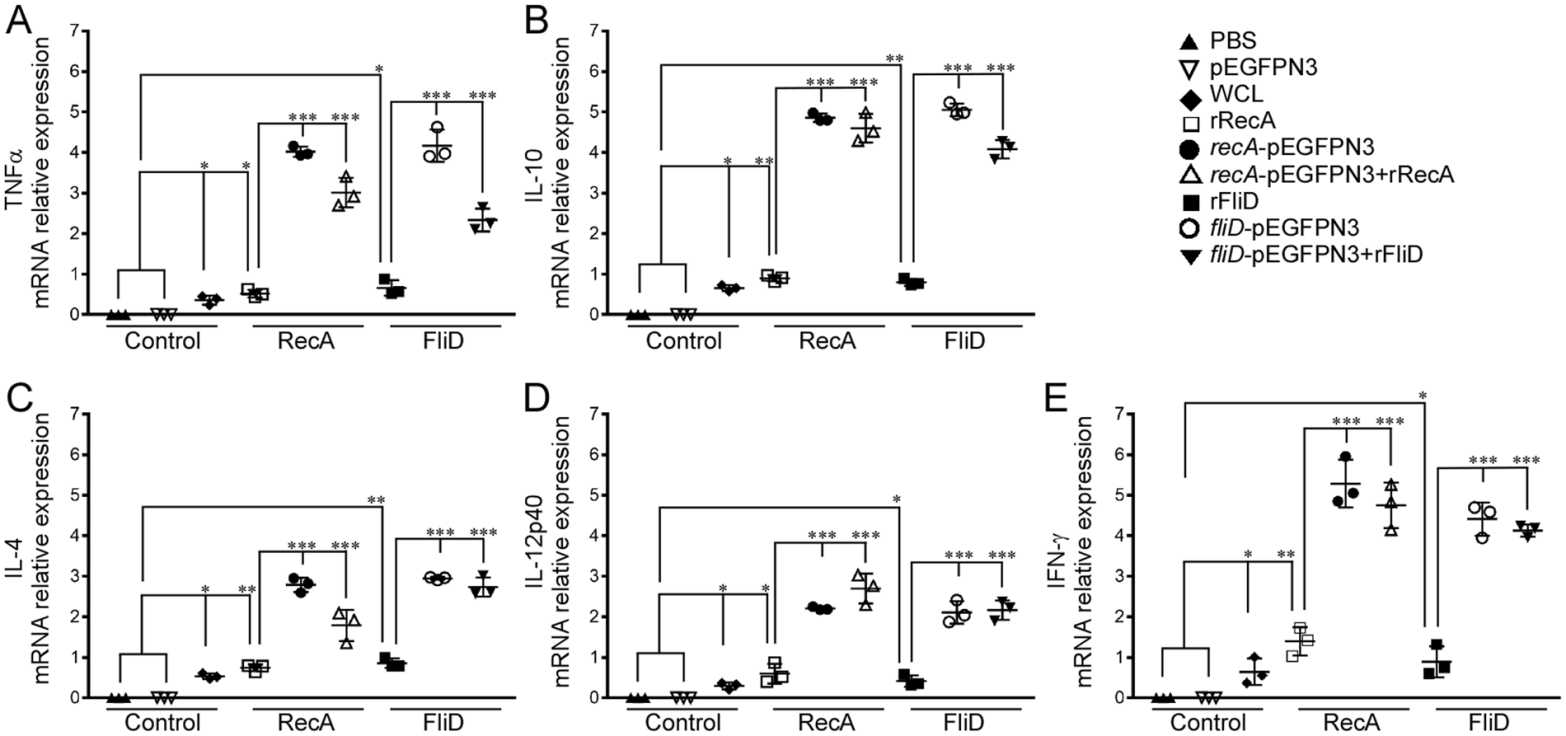
Evaluation of cell mediated immune response in control and immunized hamster groups by qRT-PCR. (**A**) TNFα, (**B**) IL-10, (**C**) IL-4, D) IL-12p40, (**E**) IFN-γ. The relative CT (ΔΔ CT) method was used to quantify cytokine gene expression: CTs were normalized against the GAPDH gene CT (Δ CT) and then compared to the same normalized gene in the PBS or pEGFPN3 immunized hamster group (calibrator). The control groups were set to 1. ns-no significance, *P < 0.05, **P < 0.01, ***P < 0.001. P values were obtained through comparison with the negative control (PBS or pEGFPN3) using a Tukey’s multiple comparisons test by 2way ANOVA.

The induction of both Th1 and Th2 type cytokines were evaluated from total mRNA isolated from spleen samples of vaccinated hamsters. mRNA abundance of Th1 and Th2 cytokines were measured by qRT-PCR analysis **(**Fig. 3**)**. The groups that received prime boost/DNA vaccines showed significant differences (*P* < 0.001) in cytokine levels when compared to control groups. In contrast, vaccination with WCL/recombinant proteins elicited cytokine response which was comparably less significant (*P* < 0.05).

### Protective efficacy of vaccines

**Figure 4:**
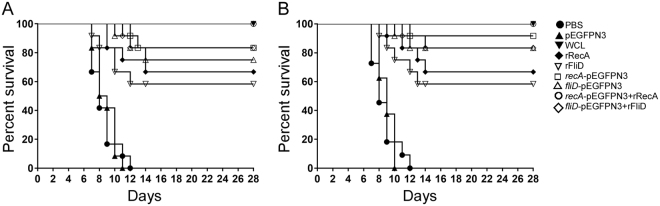
Immunoprotective potential of immunized hamster groups after challenge. (**A**) Survival graph of animals immunized with different vaccines after N2 (homologous) (**B**) PAI-1 (heterologous) lethal challenge and the survival curves were compared using log-rank analysis. Values are in Mean ± SD of different (0–28) days after challenge. Two-tailed *P* value was determined by Fisher exact test in comparison to the result of different groups observed as follows: rRecA vs *recA*-pEFGPN3 + rRecA (<0.01); *recA*-pEGFPN3 vs *recA*-pEFGPN3 + rRecA (ns); rFliD vs *fliD*-pEGFPN3 + rFliD (<0.10); *fliD*-pEGFPN3 vs *fliD*-pEGFPN3 + rFliD (ns); rRecA vs *recA*-pEGFPN3 (<0.10); rFliD vs *fliD-*pEGFPN3 (<0.10) for homologous challenge with Autumnalis N2 (**A**) and rRecA vs *recA*-pEFGPN3 + rRecA (<0.05); *recA*-pEGFPN3 vs *recA*-pEFGPN3 + rRecA (ns); rFliD vs *fliD*-pEGFPN3 + rFliD (<0.05); *fliD*-pEGFPN3 vs *fliD*-pEGFPN3 + rFliD (ns); rRecA vs *recA*-pEGFPN3 (ns); rFliD vs *fliD-*pEGFPN3 (ns) for heterologous challenge with Canicola PAI-1 (**B**). ns- Not significant (*P* > 0.01).

The protective efficacy of RecA/FliD in hamsters against homologous challenge (Autumnalis strain N2) or heterologous challenge (Canicola strain PAI-1) was determined in two independent experiments. The survival analysis in heterologous or homologous challenged hamsters showed ~58–100% survival among vaccinated animals over a period of 28 days **(**Table 1**)**. On the contrary, groups that received PBS or pEGFPN3 vector control group showed a median survival of <8 days **(**Fig. 4**)**. Among the vaccinated groups, hamsters that received DNA vaccines *recA*- pEGFPN3*/fliD*- pEGFPN3) display a protection of ∼75–83% against N2 infection (*P* < 0.01) and ∼83–91% against PAI-1 infection (*P* < 0.01). This is significantly (*P* < 0.10) higher than the protection offered by RecA recombinant protein vaccination. Though the recombinant protein vaccinated groups show ∼58–66% protection, when used along with DNA vaccines in the form of prime boost vaccine increased its efficacy to ∼91–100% (*P* < 0.01). Next, we asked whether sterilizing immunity is offered by vaccination. To test this, presence of leptospires from kidneys of the surviving hamsters were evaluated by reisolation in EMJH media. Approximately 58.4–100% of vaccinated hamsters’ kidney show sterilizing immunity with striking immunity offered by prime boost vaccination with *recA*- pEGFPN3 + rRecA (100%). Additionally, our re-isolation of leptospires were confirmed by rea-time PCR. Our 16S rRNA gene based real-time PCR analysis shows complete reduction of leptospiral DNA 28 days post-infection compared to 0 day (~10^7^ leptospires) controls (*P* < 0.001).

**Table 1:** Immunoprotective potential of different vaccine strategies after challenge.

Vaccine groups	% Protection^a^			*P* value*	Sterile immunity	
	Exp. 1	Exp. 2	Total		Culture^b^	Real-Time PCR^c^
**Autumnalis N2 - Homologous Challenge**						
*recA*-pEGFPN3 + rRecA	100 (6/6)/	100 (6/6)	100 (12/12)	<0.01	0 (0/12)	0 (0/12)
*fliD*-pEGFPN3 + rFliD	66.6 (4/6)	100 (6/6)	83.3 (10/12)	<0.01	16.6 (2/12)	25 (3/12)
*recA*-pEGFPN3	83.3 (5/6)	100 (6/6)	91.6 (11/12)	<0.01	8.3 (1/12)	16.6 (2/12)
*fliD-*pEGFPN3	66.6 (4/6)	83.3 (5/6)	75 (9/12)	<0.01	25 (3/12)	25 (3/12)
rRecA	50 (3/6)	66.6 (4/6)	58.3 (7/12)	<0.01	41.6 (5/12)	58.3 (7/12)
rFliD	50 (3/6)	33.3 (2/6)	41.6 (5/12)	ns	58.3 (7/12)	58.3 (7/12)
WCL	100 (6/6)	100 (6/6)	100 (12/12)	<0.01	0 (0/12)	0 (0/12)
pEGFPN3	0 (0/6)	0 (0/6)	0 (0/12)	ns	100 (12/12)	100 (12/12)
PBS	0 (0/6)	0 (0/6)	0 (0/12)	ND	100 (12/12)	100 (12/12)
**Canicola PAI 1 - Heterologous Challenge**						
*recA*-pEGFPN3 + rRecA	100 (6/6)/	100 (6/6)	100 (12/12)	<0.01	0 (0/12)	0 (0/12)
*fliD*-pEGFPN3 + rFliD	83.3 (5/6)	83.3 (5/6)	83.3 (10/12)	<0.01	16.6 (2/12)	16.6 (2/12)
*recA*-pEGFPN3	100 (6/6)	83.3 (5/6)	91.6 (11/12)	<0.01	16.6 (2/12)	25 (3/12)
*fliD-*pEGFPN3	83.3 (5/6)	83.3 (5/6)	83.3 (10/12)	<0.01	33.3 (4/12)	41.6 (5/12)
rRecA	50 (3/6)	83.4 (5/6)	66.7 (8/12)	<0.01	33.3 (4/12)	50 (6/12)
rFliD	66.7 (4/6)	50 (3/6)	58.3 (7/12)	<0.01	41.6 (5/12)	50 (6/12)
WCL	100 (6/6)	100 (6/6)	100 (12/12)	<0.01	0 (0/12)	0 (0/12)
pEGFPN3	0 (0/6)	0 (0/6)	0 (0/12)	ns	100 (12/12)	100 (12/12)
PBS	0 (0/6)	0 (0/6)	0 (0/12)	ND	100 (12/12)	100 (12/12)

Exp. 1 – Experiment 1, Exp. 2 – Experiment 2, *Two-tailed *P* value was determined by Fisher exact test in comparison to the result for the PBS group; ns- Not significant (*P* > 0.01); ND - Not determined; ^a^Protection in %, (the number of survivors/total challenged), ^b^Culture, % reisolation from kidney (Number positive/total number tested), ^c^16S rRNA gene based real-time PCR from kidney tissues of hamsters (Number positive/total number tested).

## Discussion

The currently available vaccines for leptospirosis are far behind, due to their inadequacy to protect the animals from different serovars confronted from various animal and environmental sources^31^. In concise, they indeed lack cross clade protection from one serovar to another serovar. Humans are the prime susceptible host for leptospirosis and used to consociate leptospirosis through infected animal sources. Especially the pet animals like the dogs and the domesticated cattle are reported to be the major source for human infection apart from their recreational and other environmental activities^32^. Habitually, the domesticated animals are the transient carriers and their incidence is through wild rodents. Predominantly, wild rodents are reported to be a carrier for serovar Autumnalis and the canine for Canicola. Consequently, in the current scenario in developing countries any vaccine candidate should offer protection against Canicola and Autumnalis infection and cross clade protection. Considering these actualities, we have investigated the prevalence of leptospirosis among canine population, which has evidenced an overall seroprevalence of 37.7% (51/135) with the predominant serovar to be Autumnalis 9.6%, followed by Canicola 8.14%^33^. Next, we assessed the efficiency of the present day available vaccine (MEGAVAC-6). The humoral immune response of the Megavac-6 vaccinated dogs against the leptospiral whole cell lysate, lipopolysaccharide (LPS), recombinant LipL32, rGroEL, rLK73.5 and rLigA were assessed in different time intervals. Among all the antigens the whole cell sonicated antigens followed by leptospiral LPS exhibited the highest antibody production among the vaccinated dogs after 64 days from the initial vaccination and drops down after 9 months. This may be due to the dominance of serovar specific leptospiral LPS, which may be the dominant immunogenic antigen in inactivated bivalent leptospiral vaccines^34^.

Although vast information is available about the immunogenic proteins of *Leptospira*^35^, especially the outer surface membrane proteins are of great interest, and several studies have demonstrated their potential uses in vaccine development^7^. Among them Lig (Leptospiral immunoglobulin-like) family proteins are well-studied with proven vaccine efficacy to be 90–100% in case of both recombinant protein and as DNA vaccination^36–38^. However, recent studies reported failure of complete protection to animals using Lig family proteins in addition to other surface leptospiral proteins^39^. The lack of success in developing vaccination was due to the following reasons: (1) lack of protein conservation among all pathogenic leptospiral species^40^, (2) lack of sterilizing immunity^36^, (3) promising model animals to perform leptospiral vaccination study (survival of control animals in challenge experiments were demonstrated^14^, and finally (4) lack of cross-clade protection. This prompted us to identify the intensive vaccine candidates, which might show a cross clade protection for leptospirosis. Adopting an expression gene library screen of virulent *Leptospira interrogans* serovar Autumnalis strain N2, we identified RecA and FliD with unique antigenic properties^15^. Apparently, these proteins were expressed only during natural infection of the host and remained undetectable in *in vitro* cultures. We hypothesized targeting *in vivo* expressed proteins as vaccine candidate may offer warranted protection against leptospiral infection. The high immunogenic nature (Fig. 1) and the conservation of these proteins among pathogenic leptospires (Fig. S1) prompted us to develop a DNA vaccine using RecA and FliD.

In the present study, the humoral immune response developed with DNA vaccination was significantly lower when compared to immunization with recombinant proteins (Fig. 2). On the contrary, in challenge experiments, DNA vaccination offered more protection both against homologous and heterologous infection (Fig. 4). Remarkably, high level of protection was conferred by prime boost vaccination reaching a survival percentage between 91 and 100%. Though animals immunized with the recombinant proteins display increased IgG response, only 58–66% of animals survived against the challenge. The reciprocal relationship between the humoral immune response and survivability, predicts humoral immune response alone is not sufficient to eliminate these spirochetes from the host. Additionally, the positive correlation established in the present between the cytokine levels in vaccinated groups and survivability substantiated an indispensable role for cell-mediated immunity (CMI) to clear pathogens from the host system. Our results are in compliance with previous report where the outer membrane leptospiral protein LipL32 impart protection when used as a DNA vaccine but failed to do so in the form of recombinant protein^41,42^. Hence, an effective leptospiral vaccine must be a vital combination option that would not only prevent invasion of spirochetes but also should eliminate those that somehow escaped the immune response and have formed successful niche in various organs.

All animals in the non-vaccinated groups died before day 12 in the present study, which shows the virulence of the strain, and the potential of the designed experimental animal model. The key success of vaccine development is to achieve sterilizing immunity and to confer heterologous protection. Majority of vaccine candidates available till data fail to offer sterilizing immunity^7^ against leptospiral infection. Because, leptospirosis spreads through shedding of leptospires in urine of carrier animals^43^. Therefore, use of effective vaccine candidates that provide sterilizing immunity could have more prophylactic applications for the prevention of leptospirosis. Though sterilizing immunity was offered by LigB (131–645) subunit vaccine, heterologous protection was overlooked in the study making it very preliminary^44^. Interestingly in our present study, the prime boost vaccine conferred protection in heterologous challenge with sterilizing immunity. Being an *in vivo* expressed protein, the use of *recA* or *fliD* as a prime boost vaccine candidate could have a promising role in reducing the disease burden of leptospirosis. Due to their effective protection against homologous and heterologous challenge infection these candidates are valued for protection of domesticated animals from Autumnalis and Canicola infection, a common threat from rodent carriers.

In an earlier study, the TcG2/TcG4 DNA prime boost vaccine provided protective immunity against *Trypanosoma cruzi* infection and Chagas disease^45^. Apart from eliciting a strong immune response, sterilizing immunity was achieved in RecA prime-boost immunized hamsters. This is considered as an important finding of our study, as sterilizing immunity as most vaccine candidates failed to induce sterilizing immunity against leptospirosis^6,38^. Hamsters immunized with recombinant *Mycobacterium bovis* BCG expressing LipL32 or LigBrep-based prime-boost vaccination strategy conferred high-level of immunization and sterilizing protection against lethal infection in the hamster model of leptospirosis^46,47^.

The possible explanation for achieving such high efficacy in the heterologous prime-boost vaccine was due to development of antigen-specific antibodies and type 1/2 cytokines against challenge infection. Earlier investigations evidenced that the enhanced efficacy of a prime-boost approach could probable be a synergistic effect in which the DNA vaccines elicits robust T-cell responses that are critical for the development of T-cell-dependent antibody responses, and in priming antigen-specific memory B cells^48,49^. Further the delivery of recombinant proteins as boosting dose effectively stimulate antigen-specific memory B cells to differentiate into antibody-secreting cells thus resulting in production of high-titer, antigen-specific antibodies^50^. To the best of our knowledge, this is the first report documenting a prime-boost vaccination strategy that provides significant protection against homologous (Autumnalis strain N2) and cross-clade heterologous (Canicola strain PAI-1) challenge infections.

In conclusion, our outcomes further demonstrate that *recA* or *fliD* prime boost vaccine could impart a significant level of protection as revealed by enhanced survival, increased humoral and cell mediated response and sterilizing immunity after challenge. Finally, we conclude that our *in vivo* antigen based DNA prime protein boost vaccine can activate a strong humoral and CMI thus seems to be an ideal vaccine candidate for leptospirosis and may be a better control of zoonotic transmission to humans.

## Electronic supplementary material

Supplementary Materials
