# Supplementary material for: Heterologous DNA prime-protein boost immunization with RecA and FliD offers cross-clade protection against leptospiral infection
Source: Sci Rep. 2018 Apr 24;8:6447. doi: 10.1038/s41598-018-24674-8 (PMC5915591; doi:10.1038/s41598-018-24674-8)
Supplement: Supplementary file 1 — Supplementary Materials [file 41598_2018_24674_MOESM1_ESM.docx]

**Supplementary Information**

**Heterologous DNA prime-protein boost immunization with RecA and FliD offers cross-clade protection against leptospiral infection**

Veerapandian Raja^1#^, Sankaran Sobana^1#^, Charles Solomon Akino Mercy^1^, Bianca Cotto^2^, Durlav Prasad Bora^3^, Kalimuthusamy Natarajaseenivasan^1*^

**Affiliation:**

^1^Medical Microbiology Laboratory, Department of Microbiology, Centre of Excellence in Life Sciences, Bharathidasan University, Tiruchirappalli 620 024, Tamilnadu, India.

^2^Lewis Katz School of Medicine, Temple University, Philadelphia, PA 19140, USA

^3^Department of Microbiology, College of Veterinary Science, Assam Agricultural University, Guwahati 781022, Assam, India

#Authors contributed equally

**Table S1. Primers used for DNA vaccine construction**

| **Gene name** | **Direction** | **Sequence (5’-3’)** | **Restriction site** | **Product Size (bp)** |
| --- | --- | --- | --- | --- |
| *recA* | Forward | GCGCTCGAGATGGGAGAAAGTATCATGAAGAAA | *XhoI* | 1101 |
|  | Reverse | GCGAATTCGCCTGCGGCCTGTTCTAAT | *EcoRI* |  |
| *fliD* | Forward | CCAAGCTTATGCCCGCGTTTACGATT | *HindIII* | 1920 |
|  | Reverse | GCGGATCCCTCGTTTCTAGCGCCTTTTA | *BamHI* |  |

***Note:*** Underlined sequences denote restriction sites.

**Table S2. Primers used for qRT-PCR**

| **Gene**  **name** | **Description** | **Direction** | **Sequence (5’-3’)** | **Product Size (bp)** | **Reference** |
| --- | --- | --- | --- | --- | --- |
| TNFα | Tumor necrosis factor alpha | Forward | AACGGCATGTCTCTCAA | 278 | 28 |
|  |  | Reverse | AGTCGGTCACCTTTCT |  |  |
| IFN-γ | Interferon gamma | Forward | GACAACCAGGCCATCC | 226 | 28 |
|  |  | Reverse | CAAAACAGCACCGACT |  |  |
| IL-4 | Interleukin -4 | Forward | CTCCTATCACTGACGGT | 342 | 28 |
|  |  | Reverse | ATTCACATTGCAGCTCT |  |  |
| IL-10 | Interleukin -10 | Forward | TGGACAACATACTACTCACTG | 308 | 28 |
|  |  | Reverse | GATGTCAAATTCATTCATGGC |  |  |
| IL-12p40 | Interleukin-12p40 | Forward | AGATCCTAAAAATAAGACCTT | 308 | 28 |
|  |  | Reverse | AGTTCTCGTATTTATACTTGT |  |  |
| β-Actin | Beta actin | Forward | TCTACAACGAGCTGCG | 357 | 28 |
|  |  | Reverse | CAATTTCCCTCTCGGC |  |  |
| 16S rRNA | Leptospiral 16S ribosomal RNA | Forward | CCCGCGTCCGATTAG | 87 | 29 |
|  |  | Reverse | TCCATTGTGGCCGRA/GACAC |  |  |

**Table S3: Prediction of B-cell epitopes based on BCPred score**

| **Protein** | **Epitopes** | **Epitopes ranked based on** **BCPred score** | **BCPRED score^A^** | **VaxiJen score^B^** |
| --- | --- | --- | --- | --- |
| RecA | Epitope 1 | P_345_LLVQENNKKSRKEEKLEQA_364_ | 0.998 | 1.3418 |
|  | Epitope 2 | R_241_KIETIKEKEESVGNRVRVK_260_ | 0.998 | 1.2859 |
|  | Epitope 3 | W_305_YSYNTEKIGQGKEAAKEYL_324_ | 0.986 | 0.8121 |
|  | Epitope 4 | M_1_GESIMKKAKEDAPSVDDSK_19_ | 0.869 | 0.6003 |
| FliD | Epitope 1 | A_436_VDKNSTVRDGKEEGGEIGQ_455_ | 0.999 | 1.5801 |
|  | Epitope 2 | S_85_GDASRSASSGKRRIEIKEL_104_ | 0.991 | 1.4418 |
|  | Epitope 3 | I_502_GINTGKVGSKWADIQDGFL_521_ | 0.948 | 0.6937 |
|  | Epitope 4 | G_479_MKTVASSSYPVSGENSVRM_498_ | 0.940 | 0.9569 |
|  | Epitope 5 | N_393_LHKKTEGPVNIDIKTDSDK_412_ | 0.938 | 1.2245 |
|  | Epitope 6 | E_24_LEAKPIRRLEQQNSFNKAQ_44_ | 0.854 | 0.5361 |

**^A^** **BCPRED score:** Epitopes having BCPred score >0.8

**^B^** **Vaxijen score:** Epitopes having VaxiJEN score >0.4

**Table S4: Number of predicted strong binder major histocompatibility complex-I and II (MHC-I & II) epitopes in RecA and FliD**

| **Alleles** | **RecA** | **FliD** |
| --- | --- | --- |
| MHC I |  |  |
| HLA A allelic variants |  |  |
| A01:01 | 6 | 8 |
| A26:01 | 9 | 12 |
| A02:01 | 2 | 19 |
| A02:03 | 32 | 49 |
| A32:01 | 4 | 13 |
| A02:06 | 13 | 36 |
| A68:02 | 18 | 36 |
| A23:01 | 5 | 5 |
| A24:02 | 6 | 6 |
| A03:01 | 12 | 32 |
| A11:01 | 12 | 44 |
| A30:01 | 28 | 42 |
| A30:01 | 12 | 8 |
| A31:01 | 2 | 4 |
| A68:01 | 21 | 44 |
| HLA B allelic variants | | |
| B40:01 | 16 | 31 |
| B44:02 | 16 | 43 |
| B44:03 | 13 | 40 |
| B57:01 | 1 | 9 |
| B58:01 | 2 | 9 |
| B15:01 | 5 | 19 |
| B07:02 | 5 | 7 |
| B35:01 | 6 | 13 |
| B51:01 | 7 | 15 |
| B53:01 | 5 | 8 |
| B08:01 | 5 | 3 |
| MHCII |  |  |
| DRB1/3/4/5 locus |  |  |
| DRB10401 | 0 | 18 |
| DRB10101 | 76 | 136 |
| DRB10301 | 11 | 15 |
| DRB10405 | 0 | 17 |
| DRB10701 | 32 | 40 |
| DRB10802 | 5 | 5 |
| DRB10901 | 0 | 8 |
| DRB11101 | 24 | 26 |
| DRB11302 | 46 | 15 |
| DRB11501 | 20 | 24 |
| DRB30101 | 6 | 24 |
| DRB40101 | 28 | 13 |
| DRB50101 | 28 | 69 |
| DQA1/DQB1 | | |
| DQA10501-DQB10301 | 46 | 55 |
| DQA10101-DQB10501 | 2 | 0 |
| DQA10102-DQB10602 | 12 | 22 |
| DQA10301-DQB10302 | 4 | 0 |
| DQA10401-DQB10402 | 0 | 0 |
| DQA10501-DQB10201 | 3 | 0 |
| DPA1/DPB1 locus | | |
| DPA10103-DPB10201 | 5 | 0 |
| DPA10201-DPB10101 | 13 | 4 |
| DPA10201-DPB10501 | 0 | 7 |
| DPB10301_DPB10401 | 0 | 3 |
| DPA10301-DPB10402 | 14 | 14 |

**Figure S1. A) Presence and conservation of *recA* and *fliD* in *Leptospira* spp. by PCR**

**
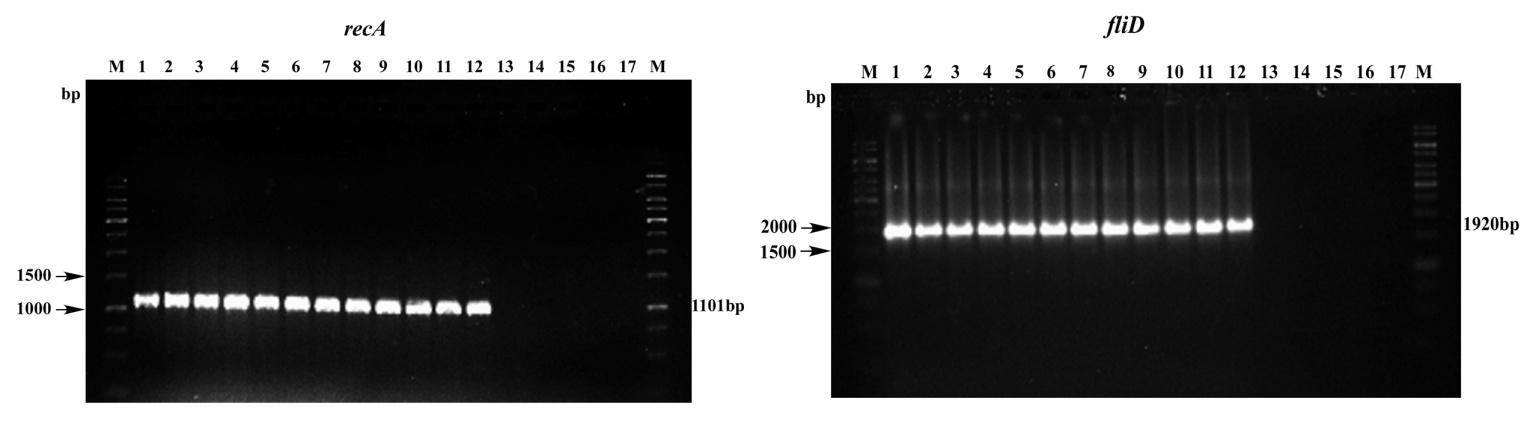
**

***Legends:*** M-1kb DNA ladder (Thermo Scientific, Rockford, IL), 1-6. *L. interrogans* (Autumnalis, Australis, Bataviae, Canicola, Pomona, Icterohaemorrhagiae), 7-8*. L. kirschneri* (Grippotyphosa, Cynopteri), 9-10. *L. borgpetersenii* (Ballum, Javanica), 11. *L. alexanderi* (Manhao), 12. *L. weilii* (Celledoni), 13-14. *L. biflexa* (Andamana, Semaranga), 15-16. Nonpathogenic strains (G3, G6), 17. Assay control.

**Figure S2. Conservation of the RecA protein with their immunogenic epitopes as determined by multiple sequence alignment among different serovars of *Leptospira.***

***Legends:***The RecA sequences of *L. interrogans* serovar Autumnalis (AGW25358), *L. interrogans* serovar Australis (EMY22543), *L. interrogans* serovar Canicola (EKO69724), *L. interrogans* serovar Copenhageni (AAS70334), *L.interrogans* serovar Icterohaemoorhagiae (EKP23302), *L. interrogans* serovar Pomona (EMF34262), *L. kirschneri* serovar Grippotyphosa (EJO70816), *L. noguchii* (WP_004424072) were retrieved from GenBank and analyzed with the CLC Main workbench (version 7.9.1.).

**Figure S3. Conservation of the FliD protein with their immunogenic epitopes in as determined by multiple sequence alignment among different serovars of *Leptospira.***

***Legends:*** The FliD sequences of *L. interrogans* serovar Autumnalis (EMN53408), *L. interrogans* serovar Australis (OOB99359), *L. interrogans* serovar Canicola (EKO71148), *L. interrogans* serovar Copenhageni (AAS69344), *L.interrogans* serovar Icterohaemoorhagiae (EMO07105), *L. interrogans* serovar Pomona (EMJ59305), *L. kirschneri* serovar Grippotyphosa (EJO70003)), *L. noguchii* (WP_004450466) were retrieved from GenBank and analyzed with the CLC Main workbench (version 7.9.1.).

**Figure S4. Restriction endonuclease analysis (REA) of DNA vaccine constructs**


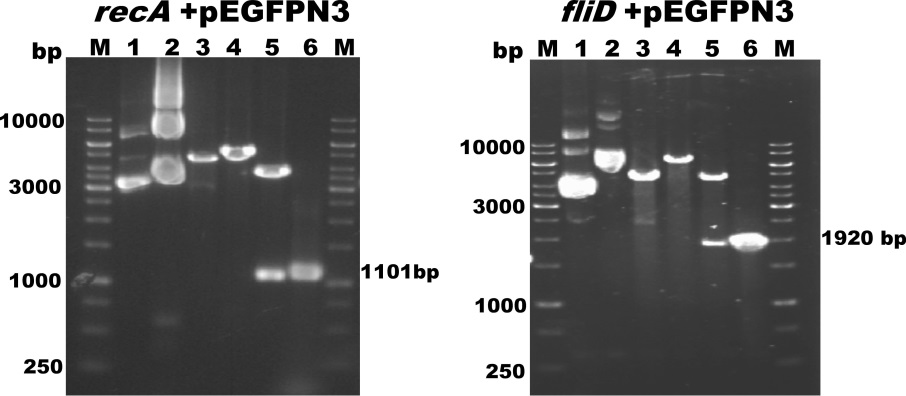


***Legends:*** M: 1 kb Ladder (Thermo Scientific, Rockford, IL), 1: Undigested pEGFPN3 vector, 2: Undigested construct (vector +gene), 3: Double digestion of vector, 4: Single digestion of construct, 5: Double digestion of construct, 6: Colony PCR product.

**Figure S5. *In vivo* expression of DNA vaccine immunized hamster groups by immunoblotting**

**
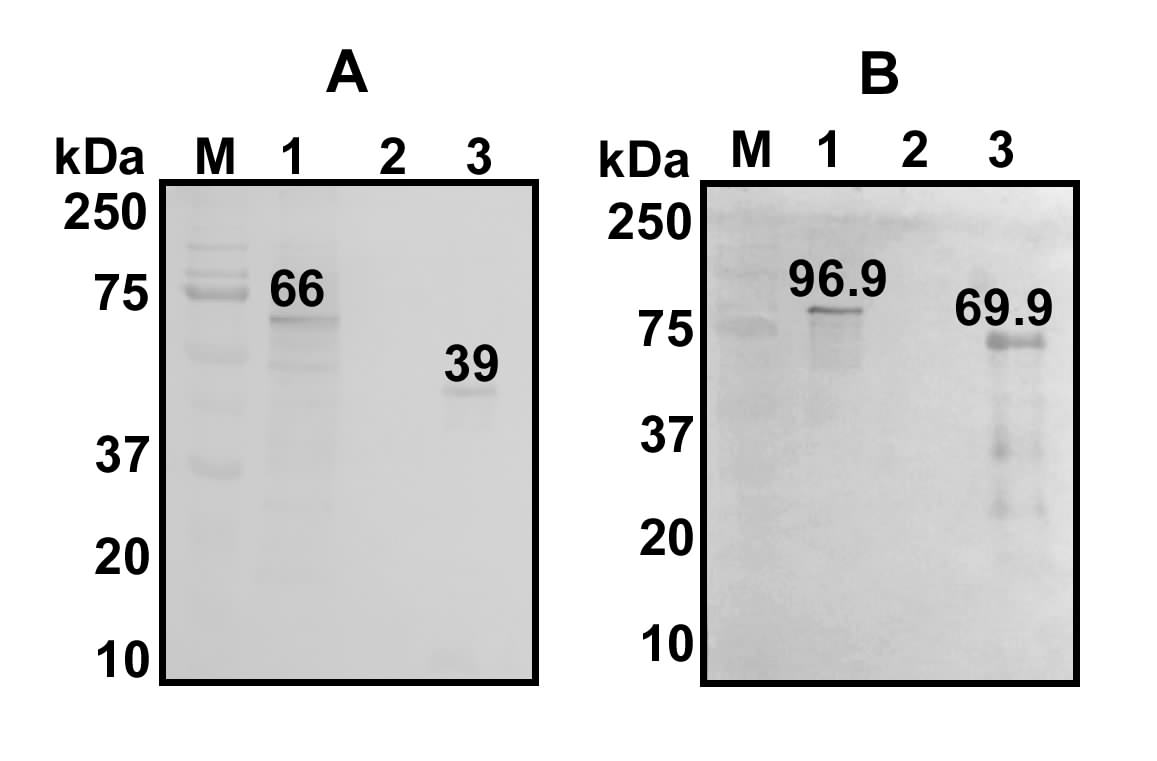
**

***Legends:*** A) *recA/*pEGFPN3 expression and B) *fliD/*pEGFPN3 expression. M- Protein ladder (Bio-Rad, Hercules, CA, USA), 1- *recA* or *fliD* DNA immunized hamster hind leg tissue, 2 – pEGFPN3 immunized hamster hind leg tissue, 3- rRecA or rFliD.

**Figure S6. Reactivity of rRecA and rFliD immune sera with homologous and heterologous rLigA and rLipL32 for 6xHis-tag cross reactivity**


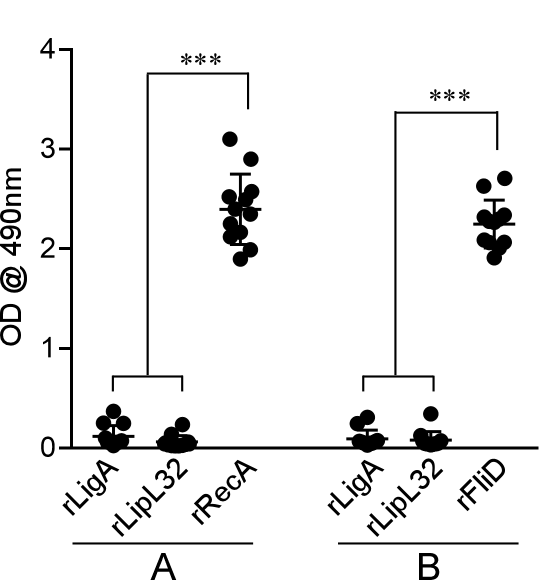


*Legends:* Reactivity of rRecA immune sera (A) and rFliD immune sera (B) against rLigA, rLipL32 and homologous antigen by ELISA. ****P*<0.001.
